# ArrayInitiative - a tool that simplifies creating custom Affymetrix CDFs

**DOI:** 10.1186/1471-2105-12-136

**Published:** 2011-05-06

**Authors:** Christopher C Overall, D Andrew Carr, Ehsan S Tabari, Kevin J Thompson, Jennifer W Weller

**Affiliations:** 1 Department of Bioinformatics and Genomics, University of North Carolina - Charlotte, 9201 University City Blvd., Charlotte, NC 28223-0001, USA; 2 Accelerated Technology Laboratories, Inc. 496 Holly Grove School Rd., West End, NC 27376, USA

## Abstract

**Background:**

Probes on a microarray represent a frozen view of a genome and are quickly outdated when new sequencing studies extend our knowledge, resulting in significant measurement error when analyzing any microarray experiment. There are several bioinformatics approaches to improve probe assignments, but without in-house programming expertise, standardizing these custom array specifications as a usable file (e.g. as Affymetrix CDFs) is difficult, owing mostly to the complexity of the specification file format. However, without correctly standardized files there is a significant barrier for testing competing analysis approaches since this file is one of the required inputs for many commonly used algorithms. The need to test combinations of probe assignments and analysis algorithms led us to develop ArrayInitiative, a tool for creating and managing custom array specifications.

**Results:**

ArrayInitiative is a standalone, cross-platform, rich client desktop application for creating correctly formatted, custom versions of manufacturer-provided (default) array specifications, requiring only minimal knowledge of the array specification rules and file formats. Users can import default array specifications, import probe sequences for a default array specification, design and import a custom array specification, export any array specification to multiple output formats, export the probe sequences for any array specification and browse high-level information about the microarray, such as version and number of probes. The initial release of ArrayInitiative supports the Affymetrix 3' IVT expression arrays we currently analyze, but as an open source application, we hope that others will contribute modules for other platforms.

**Conclusions:**

ArrayInitiative allows researchers to create new array specifications, in a standard format, based upon their own requirements. This makes it easier to test competing design and analysis strategies that depend on probe definitions. Since the custom array specifications are easily exported to the manufacturer's standard format, researchers can analyze these customized microarray experiments using established software tools, such as those available in Bioconductor.

## Background

DNA microarrays are a ubiquitous platform, the original form of highly multiplexed assays for conducting genome-wide experiments. The arrays are designed as two-dimensional grids of oligonucleotides ('probes'), affixed to a solid support at a specific location ('feature') via spotting or direct synthesis. When a solution of some labeled, purified cellular fraction ('targets') — most often polynucleotides — is applied to the array, a stable interaction forms between those subsets of probes and targets sharing sufficiently complementary regions. After the required reaction time, unbound target is washed from the array and then a scanner captures the induced signal emitted by the bound molecules. The resulting image file is ultimately used as the baseline measurement for a multitude of sophisticated standardization, normalization and statistical techniques whose goal is to infer the amount of bound target as a function of a feature's intensity.

Although DNA microarrays offer a powerful method for high-throughput molecular profiling, it is difficult to reproduce experimental measurements between platforms, to determine the magnitude of target abundance and to detect low-abundance target molecules [1]. Several sources of systematic error contribute to this problem, including incorrect array design [1], batch effects [2] and instrument limitations or error [3,4]. Researchers have developed a number of statistical techniques to minimize non-biological measurement variation resulting from all of these types of systematic error, including RMA (and its gcRMA variant), dChip and MAS 5.0 [5-8]. However, these statistical techniques tend to be general rather than specific in identifying or modelling processes that affect hybridization. Since the sensitivity and specificity of a probe's hybridization affinity to a target dramatically changes its scanned intensity, and since the probe sequences on the array cannot be changed [9-13] after it is manufactured, it is of utmost importance to use the most current information when interpreting intensity values for each feature on the array. Naturally, our understanding of what probes have been influenced changes as the annotations for an organism's genome evolve. Researchers have identified probes on various arrays whose sequences show different types of hybridization problems, such as interfering secondary structure in either component, probe misassignment, and several categories of cross-hybridization [9-16]. Once identified, some researchers modify the array's specification file so that their removal or reassignment can be easily propagated to new analyses, but most simply explain how to identify them. Because different microarray platforms have unique design and construction features, as well as custom software for communicating how a probe is to be interpreted with respect to the target genome, the strategy for modifying the specification file for a given type of DNA microarray requires platform-specific strategies.

The most complex and high-density DNA microarray designs come from the Affymetrix platform products. Their complexity results from the promiscuous placement of probes with respect to target elements and the multiplicity of probes per biological target. Their high density means that there are hundreds of thousands to millions of simultaneous measurements to be considered. There are other unique design features of these arrays: the probes are relatively short oligonucleotides (25-33 nt) synthesized directly onto the array; for several generations of the 3' expression arrays, the basic measurement 'unit' was determined by a probe pair, consisting of a perfect match (PM) and a mismatch (MM) probe, although on some of the latest designs this has changed; finally, there is the conceptual grouping of probes into a probe set whose members interrogate a specific, and longer, target molecule. A probe set might discriminate among known variants of a transcript, exon, or SNP location; depending on the platform the number of probes in a probe set varies. The specification file for Affymetrix arrays is called a Chip Definition File (CDF) and delineates which, and how, probes are grouped into probe sets. There is a great deal of public data and software available for these arrays, so solutions to the problems they present have the potential for broad impact.

The advantage to such a design is the redundancy of the measurements: probe or target characteristics that confound measurements are easily remedied by removing or reassigning problematic probes from a probe set while leaving probes that faithfully report on the original target of interest. In fact, shortly after Affymetrix released the sequence information for their arrays into the public domain, several researchers analyzed the probe set definitions [15,17-22], identifying a number of potential problems with the original definitions that could produce measurement error within a probe set. They then proposed several bioinformatics methods for re-defining the probe sets to solve these problems (e.g. creating a custom array specification), intending to reduce the measurement error and to make the aggregated measurements more biologically relevant. In many cases, these groups validated their re-definition strategy by showing that their custom probe set definitions, when compared to the Affymetrix default, significantly changed the differential expression results. In some cases, subsequent studies showed that the re-definition strategy significantly improved the correlation between microarray measurements and experimental results. The custom probe set definitions of Dai *et al*. [18], and two later studies using them [23,24], illustrate how custom array specifications can significantly improve microarray measurements and the conclusions drawn from them.

For several Affymetrix expression arrays, Dai *et al*. [18] re-defined the original probe sets into gene-, transcript- and exon-specific probe sets. They used the most up-to-date versions of several public genome databases, such as UniGene [25] and Refseq [25], in this process, and then created custom CDFs for each source. In one case, they used an updated version of UniGene to define a gene-specific CDF for the Affymetrix HG-U133A chip and then reanalyzed data from a cardiac tissue study (GSE974) [26]; comparing the updated CDF and the original CDF, they found between 30-40% differences in those genes predicted to be significantly differentially expressed between the two. When performing a similar analysis with other custom CDFs, they found between 30-50% differences in predicted differential expression. Subsequently, Sandberg *et al*. [24] showed that Dai's custom probe set definitions, when compared to the original definitions, improved the accuracy and precision of transcript estimates for a set of cross-lab replicate arrays [27]. In particular, their accuracy metrics showed that the microarray measurements became more similar to those measured by RT-PCR. Later, Mieczkowski *et al*. [23] showed that Dai's custom CDFs significantly improved the correlation between microarray expression profiles and RT-PCR expression profiles. Thus, re-defining array specifications can potentially improve the down-stream analysis of Affymetrix microarrays. However, biological researchers who want to test, or simply adopt, new probeset definitions, are likely to be hindered by the way the methods are communicated.

These researchers communicated their re-definition strategies in a variety of ways. Some of them only published their *general strategies *for re-defining the probe sets, without providing custom specifications for individual microarrays; others published custom array specifications for a limited subset of microarrays, although in a file format different from the standard CDF format; still others provided custom CDFs, but again, for a limited subset of microarrays. For those research groups who can simply use a provided custom CDF, this bewildering variety of formats does not pose a problem. However, it is a problem for those groups who are not in this fortunate situation: those who want to use a published re-definition strategy, but don't have access to a custom array specification file (non-standard or standard); those who want to modify an existing method; those who want to combine multiple re-definition strategies; and those who want to develop and implement their own re-definition methods. For example, one of our research interests is to test different gene models by assigning probes to transcript-specific sets and then creating model-specific CDFs. What are the options for these researchers?

One option is to create custom versions of the algorithms for summarizing probe set intensities, such as RMA. However, writing these custom algorithms is likely to be daunting, error-prone, and hard to test. A better option is to create a custom CDF. Researchers can then generate summarized probe set intensities using any of the well-accepted and tested analysis packages provided by Affymetrix or Bioconductor [28]. Though easier, creating a custom CDF still presents challenges. In the worst case, creating a custom CDF from scratch, researchers need to thoroughly understand the file formats (ASCII, XDA) and platform-specific logical rules for defining probe sets (3' expression arrays vs. exon arrays vs. SNP arrays) necessary to parse and write CDFs. Using an existing application programming interface (API) or software development kit (SDK), such as Affymetrix's Fusion SDK [29] or *affxparser *[30] (an R wrapper of the Fusion SDK), is an easier and more efficient solution than writing in-house methods for reading and writing CDFs. However, this still requires a degree of proficiency in a specific programming language (C or Java for the Fusion SDK, R for *affxparser*), knowledge of the CDF file formats and probe set construction rules, and knowledge of the language-specific data structures for representing a CDF. A lab with in-house programming resources may opt for either of these viable approaches, but it is not ideal for labs with minimal programming expertise or those not wanting to immerse themselves in CDF minutiae. They need a new set of tools that makes creating a custom array specification easy and unambiguous.

It is for this group of biological researchers that we developed ArrayInitiative: a standalone, cross-platform desktop application for creating and managing custom versions of manufacturer-provided (default) microarray specification files, such as a CDF, and for generating easily understandable, non-standard CDF representations. It requires only minimal knowledge of array specification standards (file formats and logical rules) and zero programming expertise. The manufacturer's array specification file format is completely hidden from ArrayInitiative users, and they need only understand the most abstract notion of array organization for an array type. As such, ArrayInitiative users only have to understand and create a simple file (delimited or XML) to define their own custom array specifications. For example, when creating a custom Affymetrix 3' IVT expression array, users only need to understand that a probe set contains pairs of perfect match and mismatch probes and be able to create a minimal text-based representation of probe set membership. ArrayInitiative greatly simplifies the task of creating custom array specifications, allowing labs with less computational expertise to test, use, tweak and invent alternative methods for re-defining microarray specifications.

## Implementation

We developed ArrayInitiative as a standalone, rich client desktop application with an integrated backend database. The user interface was implemented with PyQt [31], a Python [32] binding of QT from Riverbank Computing. We used SQLite [33] as the backend database, as implemented in Python's *sqlite3 *module [34], because it requires minimal installation/setup, administration and maintenance tasks for the user and is a standard library module in Python 2.5+. Each of the main components is cross-platform and freely available. ArrayInitiative can be downloaded from the "Downloads" section at http://wellerlab.uncc.edu/ArrayInitiative/index.html and as Additional file 1 in this publication.

## Results

### Application overview

ArrayInitiative is a rich client application for creating custom array specifications built upon a default array specification. The default array specification is typically the one provided by the manufacturer and the custom array specification is a user-modified version of that default. Users can: (1) import default array specifications, (2) import probe sequences for the default array specification, (3) import a custom array specification, (4) export any array specification to multiple output formats (5) export the probe sequences for any array specification and (6) browse high-level information about the array, such as version and number of probes. This release of ArrayInitiative supports Affymetrix 3' IVT expression arrays, and all of the subsequent sections will assume this type of array.

**Figure 1:**
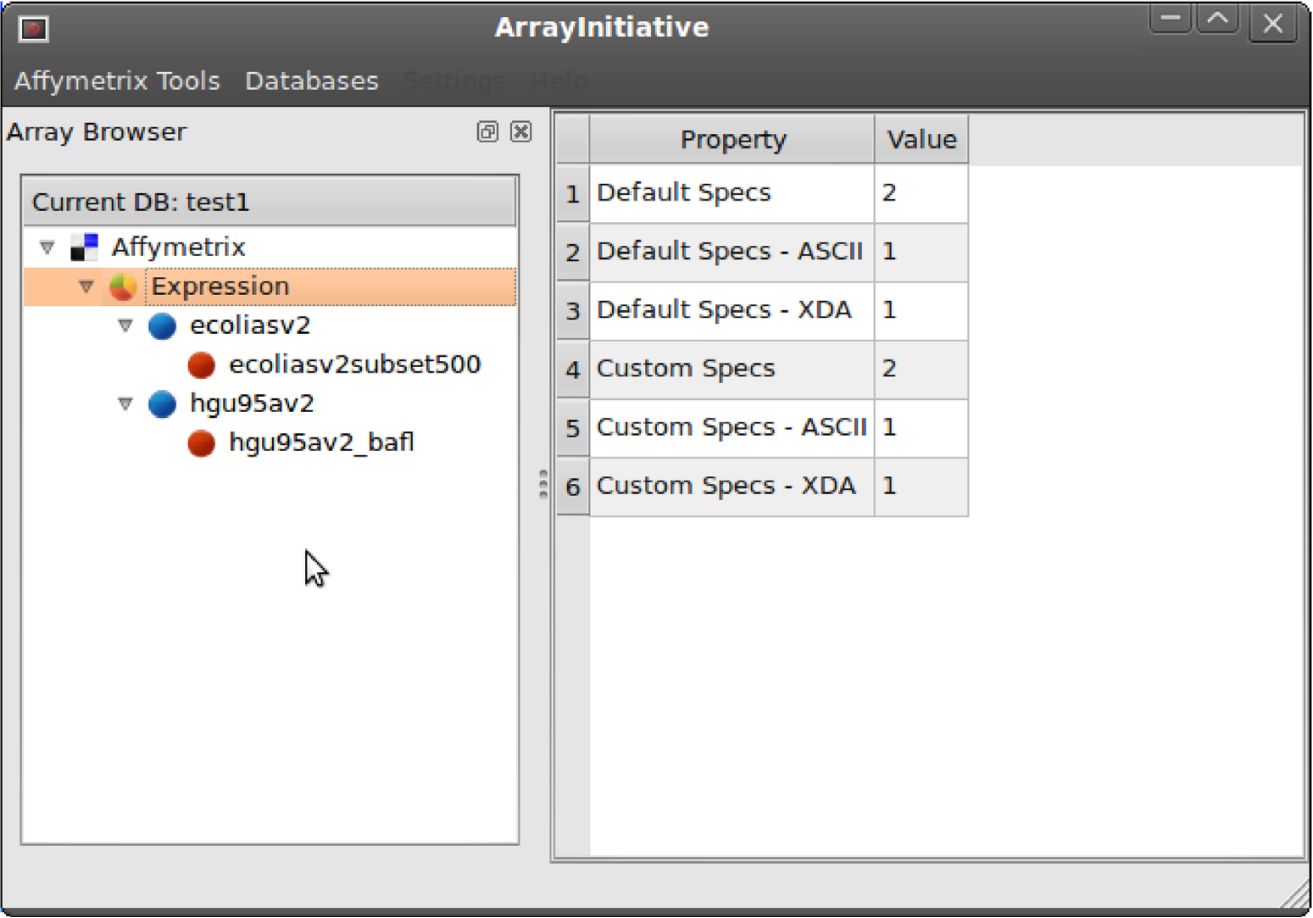
**ArrayInitiative main screen**. The ArrayInitiative main screen, consisting of an array specification browser, a dashboard and a main menu.

ArrayInitiative's default main window, shown in Figure 1, consists of an array specification browser, a dashboard and a main menu. The array specification browser displays a list of a user's array specifications, organized as a hierarchical tree, while the dashboard displays summary information about the currently selected browser item. For example, when the "Affymetrix → Expression" browser item is selected, ArrayInitiative shows how many default and custom Affymetrix 3' expression array specifications there are; when a user clicks on an array specification in the browser, summary information for that specification is displayed in the dashboard. All of ArrayInitiative's tools, such as the one for importing a default array specification, can be opened from either the main menu or from context-sensitive (right-click) menus available in the specification browser. Each of the tools in ArrayInitiative open as modal dialog windows.

### Context-sensitive (right-click) menus

The array specification browser gives right-click access to the main menu items; the resulting form values are pre-populated based on the current browser selection. Renaming and deleting array specifications can only be done using the context menu.

### Creating and managing multiple ArrayInitiative databases

When first using ArrayInitiative, users will need to create at least one database before they can access any of the array-specific functionality of ArrayInitiative. Multiple ArrayInitiative databases can be created to logically separate distinct sets of arrays, if desired. In addition, users can update the information for an existing database and switch between databases by setting the active database.

### Importing a default array specification

Users can import the array specification (probe set definitions) for an array from a CDF file (ASCII, versions 3-5 and XDA, versions 1-3). Usually, users will import a default array specification from a CDF provided by Affymetrix, but they can also import a default array specification from a custom CDF instead. Users must import at least one default array specification before importing custom array specifications and writing custom CDFs.

### Importing probe sequences for the default array specification

After importing a default array specification, users can import the probe sequences for a default array specification, using the FASTA or tab-delimited probe sequence file provided by Affymetrix for that array. ArrayInitiative will automatically generate the missing mismatch probe sequences. See the "File Formats" section of the manual — available online in the supplementary site — for details about the supported formats for a probe sequences file.

### Creating a custom array specification file

After importing a default array specification, users can create a custom array specification for any imported default array specification. When creating a custom array specification file to import, users can instruct ArrayInitiative to copy an existing probe set, re-define an existing probe set or define an entirely new probe set. When defining, or re-defining, a probe set, users can use any of the probe pairs from the default array specification. ArrayInitiative treats probe pairs as atomic units, and as such, users can't add just the PM or MM probes to a probe set definition. Currently, ArrayInitiative accepts a full specification file type (delimited or XML), requiring that users explicitly define every probe set. See the "File Formats" section of the manual — available online in the supplementary site — for details about the supported formats of a custom array specification.

### Importing a custom array specification

After creating a full specification file, users can import them into ArrayInitiative. Users can define multiple custom versions for any default array specification.

### Exporting an array specification

Users can export default and custom array specifications as a CDF (ASCII or XDA), an XML file or a delimited file. See the "File Formats" section of the manual — available online in the supplementary site — for details about the output types.

### Exporting probe sequences for an array specification

Users can export the probe sequences for a default or custom array specification as a FASTA, XML or delimited file. See the File Formats page for details about the output types. When exporting a custom array specification as a CDF, the type — ASCII or XDA — will be the same as the parent default array specification.

## Discussion

In this section, we illustrate why ArrayInitiative is useful to microarray researchers by presenting a case study in which we create custom CDFs based upon two different, published probe-filtering techniques and then use Bioconductor algorithms to investigate the effect of the probe set re-definitions on the summarized expression values. The complete case study code, data and results are available under the "Downloads" section at http://wellerlab.uncc.edu/ArrayInitiative/index.html. We then discuss the limitations and future directions of ArrayInitiative.

## Case Study

### Introduction

Imagine that you, as a researcher who is reasonably proficient with programming, discover two different probe-filtering techniques for Affymetrix arrays while reading the literature. Both of them seem reasonable and you think that, by incorporating such QC steps, you could improve the results you get when analyzing your expression arrays with Bioconductor tools. Since your favorite Bioconductor packages require a well-formed CDF, you search the web to see if someone has created a custom CDF based upon both filters. Unfortunately, you can't find one and must generate the custom CDF yourself or rewrite and test several complicated algorithms. Convinced that the filters will improve your results, you decide to create a custom CDF from scratch. The developers of technique A conveniently provide a comma-delimited text file with the new probe set definitions for the array type that you're interested in, while the developers of technique B provide a custom CDF with their filter, also for your array type. You then need to compare the two different probe set definitions to make sure they don't conflict and then merge their individual probe set definitions into a single custom CDF. Examining the delimited files is relatively straightforward, so filter A's probe set definitions are already usable; however, to get the probe set definitions for filter B, you need to parse the rather complex CDF file. After some time and effort, you manage to learn the CDF format and successfully retrieve the probe set definitions for filter B. With some coding magic, you create a joint probe set definition that is the intersection of the two filters. Confident in your knowledge of the CDF format, you write some code to create the custom CDF, which eventually is accepted by the analysis packages after much trial-and-error. Upon analyzing your arrays, it appears that, indeed, the two filtering techniques, in tandem, significantly improve your results. Excited by your success, you want to apply the same probe-level filters to an expanded set of arrays, some of which were done on a later version of the array. As you acquire the necessary files you realize that the later version of the array is described by a different kind of CDF, in the XDA format, which is entirely different from the CDF format that you learned. Dispiritedly, you set out to learn another format and start the process over again.

Not only is the above scenario likely, it is also fairly optimistic. Many research labs do not have the in-house computational expertise to create custom CDFs easily, nor should every lab be required to learn about the CDF formats to reap the benefits of research into probe-level filters on Affymetrix microarrays. This is exactly why a custom CDF creator like ArrayInitiative is useful.

The case study presented here illustrates the merging of two real sets of probe filters, that we term 'BaFL' and 'Upton' (described more fully below). We created custom HG-U95Av2 CDFs for each of them and then used three different Bioconductor packages — RMA, dChip and MAS 5.0 — to determine the independent and joint effect of each filter. Lest the reader be unconvinced that such filters would alter the outcome, for a given custom CDF and summarization method, we compared the probe set intensities calculated using the custom CDF to those calculated using the default CDF.

### HG-U95Av2 Microarray and the Bhattacharjee Data Set

The 'Bhattacharjee' data set, which contains data for arrays reporting on 139 distinct macro-dissected human lung adenocarcinoma samples, was assayed using 190 HG-U95Av2 arrays [35]. Of these, 47 samples had 2-4 replicate arrays (most have only two). The HG-U95Av2 array has 12,625 probe sets and 201,800 probe pairs (403,600 probes), with most probe sets having 16 probe pairs (32 probes). The full distribution of probe pairs per probe set is presented in Table S1.

For this case study, we analyzed twenty randomly selected arrays (RAND) from 190 Bhattacharjee adenocarcinoma arrays, shown in Table S2. When selecting the arrays, we excluded any arrays that exhibited array-wide technical problems, as identified by Thompson *et al*. [36], from the sample pool.

### Probe-filtering Techniques

#### BaFL

Thompson *et al*. [36] developed a "white box" pipeline - Biologically applied Filter Levels (BaFL) - to identify and filter microarray probes that are likely to report incorrect or misleading intensities based upon certain biological properties, such as the presence of SNPs in the probe sequence (e.g. as identified by AffyMapsDetector [10]), probe cross-hybridization, internal structure in either the probe or target sequence that reduces binding affinity, and probe intensities that fall outside the linear range of the scanning device [3,4]. Thompson *et al*. provided comma-delimited files of filtered (deprecated probes removed) probe set definitions for the HG-U95Av2 and HG-U133 array types.

#### Upton

Upton *et al*. [14,15] reported that probes with certain sequence motifs have intensities that are uncorrelated with the other probes in the same probe set; however, they tend to correlate well with any probes having the same sequence motif, regardless of probe set membership. In this case study, we will focus on the two major types of problematic sequence motifs identified by Upton *et al*. [15]: G-runs and primer spacers. Probes with the G-run motif, ≥ 4 Gs in a row, tend to produce consistently high intensities, with some position dependence. The primer spacer motif, CCTCC, is related to the incorporation of a T7-binding site when a mRNA is amplified during target preparation. When the target is amplified in this manner, the probe intensities tend to be higher than most, introducing a spurious correlation similar to that seen with G-runs. Since both of these sequence motifs introduce a systematic bias when summarizing probe set intensities, any probes including them should be removed from a CDF prior to calculating expression values. This is always true for the G-run motif and is true for the primer spacer motif when the target is amplified by incorporating a T7-binding site. The reports by Upton et al. provided good insights about identifying problematic probes, but they did not provide a modified CDF, a flat-file of probe set definitions nor a list of deprecated probes for a given array version.

### Are the Probe-Filtering Techniques Independent?

When different groups develop QC filters independently there may be overlap or conflicts of which they are unaware. Therefore, before proceeding with creating custom CDFs and downstream analysis, we first assessed the overlap between the BaFL and Upton filter sets to determine if they are truly independent filters.

**Figure 2:**
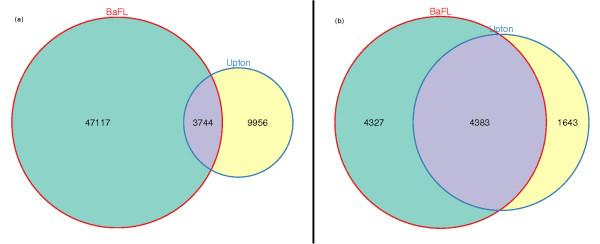
**Independent and joint effects of the BaFL and Upton filter sets**. **(a)**: The total number of probe pairs removed by either the BaFL or Upton filter sets was 56,994/201,800 (28.2%). The Venn diagram shows the number of probe pairs removed only by the BaFL filter set (blue), the number of probe pairs removed by the Upton filter set (yellow), and the number of probe pairs removed by both filter sets. **(b)**: The total number of probe sets removed or modified by either the BaFL or Upton filter sets was 9,799/12,625 (77.6%). The Venn diagram shows the number of probe sets affected only by the BaFL filter set (blue), the number of probe sets affected only by the Upton filter set (yellow), and the number of probe sets affected by both filter sets.

Figure 2a shows how many *probe pairs *were removed independently and jointly by each of the filter sets and Figure 2b shows how many *probe *sets were modified or removed independently and jointly by each of the filter sets. Most analyses are performed on probe set data, but the affected probe pairs are not necessarily homogeneously distributed, so we examined both aspects. Based upon these results, we see that the two filters generally operate on different sets of probe pairs, with minor overlap (4.1%). The BaFL filter set removes a significantly larger number of probe pairs than the Upton filter set. We also see that the two filter sets jointly affect a large fraction of the probe sets (31.1%), although a significant portion of them are independently affected by each filter. The greater overlap between the two filters in the latter case is expected since each probe set consists of multiple probe pairs.

### Creating the Custom CDFs

**Figure 3:**
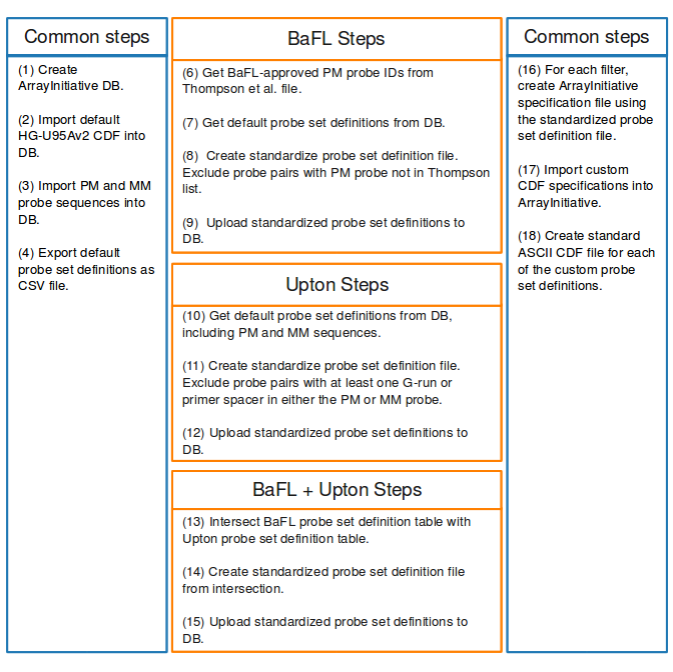
**Workflow: creating the custom CDFs**. Workflow for creating the custom BaFL, Upton and BaFL + Upton custom CDFs. The boxes in blue are common steps while the boxes in orange are steps unique to a particular filter set.

We created three custom CDFs using ArrayInitiative: a BaFL-only custom CDF, an Upton-only custom CDF and a BaFL plus Upton joint CDF. Each filter set required a unique approach for generating the probe set definitions due to the different ways that they were communicated; however, after we defined the probe sets for each filter set, the steps for creating the custom CDFs were identical. Figure 3 shows a graphical summary of the CDF creation workflow.

The first actions were common steps. We created a new ArrayInitiative database and imported the default HG-U95Av2 CDF from the file provided by Affymetrix. Next, we imported the PM probe sequences using the tab-delimited file provided by Affymetrix and instructed ArrayInitiative to automatically generate the corresponding MM probe sequences. Finally, we exported the default CDF probe set definitions (with probe sequences) as a comma-delimited text file. When generating the custom probe set definitions in the subsequent steps, we queried the ArrayInitiative database directly for information about the default probe set definitions as querying databases tends to be more efficient and straightforward than searching for information in flat files. The end-point of the custom probe set definition stage was to have in hand a comma-delimited file (CSV) with the following columns per line: (1) probe set ID, (2) PM probe ID, (3) x-coordinate of the PM probe, (4) y-coordinate of the PM probe, (5) MM probe ID, (6) x-coordinate of the MM probe and (7) y-coordinate of the MM probe. In order to keep the method comparison fair we required that each probe set have at least 4 probe pairs remaining; if it did not, we removed it before creating the custom CDFs.

When creating the BaFL probe set definitions, we started with the comma-delimited filtered probe set definitions for the HG-U95Av2 array provided by Thompson *et al*. Since that probe set definition file included only the PM probes, we first queried the ArrayInitiative database to get the full probe set definitions of the default CDF. When creating the standardized probe set definition file, we included only those probe pairs whose PM probe was in the author's probe set definitions. We then uploaded the standardized probe set definitions into the ArrayInitiative database.

Creating the Upton probe set definitions was somewhat trickier because we needed first to identify the G-run probes on the HG-U95Av2 array. Again, we first queried the ArrayInitiative database to get the full probe set definitions of the default CDF, including the PM and MM probe sequences. When creating the standardized probe set definition file for this filter, we identified probe pairs — using regular expressions — that had at least one G-run or primer spacer in either the PM or MM probe sequence and then excluded that probe pair from the final probe set definition. We then uploaded the standardized probe set definitions into the ArrayInitiative database.

Since the BaFL + Upton CDF is the intersection of the probe pairs that survived the BaFL and Upton filters, we retrieved the joint probe set definitions from the ArrayInitiative database by intersecting (standard 'INTERSECT' SQL statement) the BaFL probe set definition table and the G-run probe set definition table (created in the previous steps). Now having a list of the surviving probe pairs, we created a standardized probe set definition file and uploaded this data into the ArrayInitiative database.

Having standardized probe set definition files for each of the probe filters, the final steps for creating a custom CDF for each are identical. We first created ArrayInitiative specification files for each of the filters using the standardized probe set definition files and then imported the custom CDF specifications into ArrayInitiative. Finally, we created a standard ASCII CDF file for each of the custom probe set definitions in ArrayInitiative.

**Figure 4:**
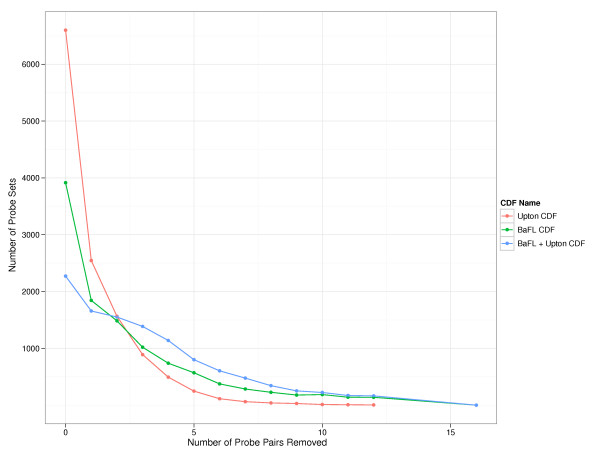
**Number of probe pairs removed by individual filter sets**. Summary of number of probe pairs removed from standard probe sets -- those having 16 probe pairs -- by each of the three filter sets. Presented for each custom CDF are the total number of probe sets that survived the cleansing process. Default CDF = 12,387 standard probe sets, Upton CDF = 12,386 standard probe sets, BaFL CDF = 10,981 standard probe sets, BaFL + Upton = 10,927 standard probe sets.

Table 1 shows how the custom CDFs were changed relative to the original and Figure 4 compares the frequency with which the indicated number of probe pairs are removed from probe sets for each of the three custom CDFs (e.g. the number of probe sets with zero probe pairs removed, one probe pair removed, two probe pairs removed, etc.)

**Table 1:** Filter set modifications to the HG-U95Av2 specification

CDF name	Removed probe sets	Modified probe sets	Unmodified probe sets
Upton filter set	1	4,083	8,303

BaFL filter set	1,406	7,125	3,856

BaFL + Upton filter set	1,460	8,570	2,357

Modifications to the default HG-U95Av2 specification made by each set of filters.

### Creating and Validating Bioconductor CDF Packages

Many of the Bioconductor packages aimed at analyzing Affymetrix arrays use a specialized R package representation of a CDF instead of the actual CDF; there are pre-generated packages for many of the default CDFs. Since we are using custom CDFs for downstream analysis, we first created and installed our own R packages for the three custom CDFs generated by ArrayInitiative, as follows:

1. Made the packages using the *make.cdf.package *function in the *makecdfenv *package [37].

2. Installed the custom CDF packages using *R CMD INSTALL*.

With the custom CDF packages successfully installed, we compared, for each filter, the probe set definitions in the existing R packages with the probe set definitions in ArrayInitiative, as follows:

1. Exported Bioconductor's internal probe set definitions for the custom CDFs - using the *ls *and *get *R functions - to a set of delimited files and then uploaded the data to the ArrayInitiative database (three tables total).

2. Verified that the number of Bioconductor probe pairs equaled the number of ArrayInitiative probe pairs (SQL 'COUNT').

3. Verified that the member probe pairs in Bioconductor were the same as the member probe pairs in ArrayInitiative (SQL 'INTERSECT').

Using the above procedure, we verified that the probe set definitions for each filter were consistent between Bioconductor and ArrayInitiative, showing that ArrayInitiative-generated CDFs are compatible with one of the most widely used microarray analysis packages. Since the probe set definitions were consistent, we can reasonably assume that any differences in downstream analysis will be the result of the custom probe set definitions, not from misinterpreting set membership.

### Differences in Summarized Probe Set Intensities

How do the BaFL and Upton filter sets independently, and jointly, affect summarized probe set expression values? For the three summarization methods chosen (MAS 5.0, dChip, and RMA), we determined how, on average, the custom expression values changed with respect to the default expression values as we removed probe pairs.

We only analyzed the 12,387 probe sets with 16 probe pairs in the default CDF (henceforth called standard probe sets) and only removed from 0 to 12 probe pairs, so that at least 4 remained to a set. We did this for several reasons: (1) standard probe sets represent the vast majority of those on the array and most are designed to interrogate transcripts, (2) the majority of non-standard probe sets represent the minority of those on the array and most are designed for diagnostic or quality control purposes and (3) we wanted to use a consistent probe set size to eliminate that as a factor when analyzing the downstream effect on expression values.

For each unique combination of summarization method and RAND array, we calculated the expression values of the standard probe sets using the default and custom probe set definitions. Then for each probe set, we calculated the percent change between the expression values, as follows:

where *E_c _*is the custom expression value and *E_d _*is the default expression value. For each distinct combination of summarization method and custom CDF, we calculated the average delta, across all of the RAND arrays, as we removed probe pairs. The workflow is depicted in Figure S1.

Before running the analysis, we postulated that the Upton filter set would decrease probe set intensities as we removed probe sets, since probes with G-runs and primer spacers tend to have a much higher intensity than other probes in the probe set; we expected that the BaFL filter set would increase the probe set expression values as we removed probe sets because its filters tend to remove low intensity PM probes; we expected that the joint filter probe set expression values would fall between those produced by the two independent filter sets, but heavily weighted towards the BaFL probe set expression values, since it removed many more probe pairs.

### MAS 5.0

**Figure 5:**
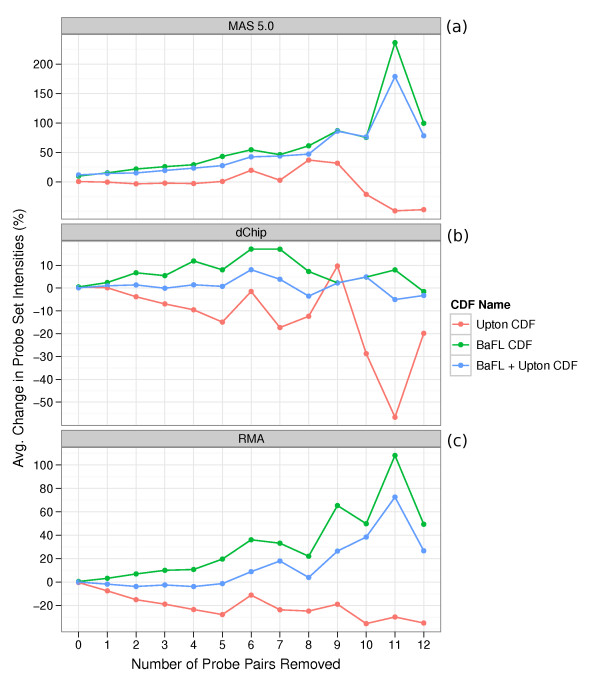
**Difference between summarized probe set intensities using the custom CDFs versus the default CDF**. Probe set intensities were summarized by MAS 5.0, dChip and RMA for each of the three custom CDFs and for the default CDF. The graphed lines show the average percent change in custom CDF probe set expression values with respect to the default CDF expression values as we removed probe pairs. **(a) **Probe set intensities were summarized by MAS 5.0. **(b) **Probe set intensities were summarized by dChip. **(c) **Probe set intensities were summarized by RMA.

Figure 5a shows the average expression changes seen when we used MAS 5.0 to summarize the probe sets. The Upton filter set influenced the probe set expression values in two distinct ways: when we removed 1-5 probe pairs, the expression values stayed relatively constant compared to the default CDF; when we removed 6-9 probe pairs, the expression values increased (except at 7); when we removed 10-12 probe pairs, the expression values decreased. This result was surprising since we expected the probe set expression values to consistently decrease. The BaFL filter set consistently resulted in increased probe set expression values as we removed probe pairs, while the joint filter set was a blend of the two independent filter sets, although heavily weighted towards the BaFL filter set.

### dChip

Figure 5b shows the average expression changes seen when we used dChip to summarize the probe sets. The Upton filter set decreased the probe set expression values, but exhibited somewhat erratic behavior. The BaFL filter set, in general, decreased the probe set expression values, reaching a maximum positive change at 6-7 probe pairs removed. The expression values decreased when we removed 12 probe pairs. The joint filter set was a blend of the two independent filter sets, only somewhat weighted towards the BaFL filter set.

### RMA

Figure 5c shows the average expression changes seen when we used RMA to summarize the probe sets. The Upton filter set consistently decreased the probe set expression values while the BaFL filter set consistently increased the expression values. The joint filter set was a blend of the two independent filter sets: the values were slightly weighted towards the BaFL filter set when we removed 1-6 probe pairs and heavily weighted towards the BaFL filter set when we removed 7-12 probe pairs.

## Case Study Discussion

The Upton filter set decreased the probe set expression values when they were summarized by dChip and RMA, a trend not observed when we summarized the probe sets with MAS 5.0. The MAS 5.0 expression values were unresponsive to the Upton filters when we removed 1-5 probe pairs, while its effect was fairly erratic in the 6-12 range. The BaFL filter set consistently increased the probe set expression values for all of the summarization methods, with MAS 5.0 and RMA being particularly responsive. The joint filter set produced intermediate expression values that were a blend of the two independent filter sets when summarized with either dChip and RMA; the effect was generally additive. The BaFL filters had a stronger influence on the expression values, but this is not surprising, given that the BaFL filter set removed significantly more probe pairs than the Upton filter set. When summarizing with MAS 5.0, changes in the expression values were largely driven by the BaFL filters, with the Upton filters having little effect.

In considering the joint filter set, RMA exhibited trends in expression value changes that best fit our prior expectations. Considering the *magnitude *of expression value changes, the joint filter changed the MAS 5.0 expression values the most, followed by RMA and then dChip. While the expression values for MAS 5.0 and RMA changed by factors of 20-100% for many of the data points, the changes seen with dChip were much lower, in the 2-15% range, suggesting that dChip is the least responsive to changes in the probe set definitions.

From these results, we may conclude that the filter sets significantly alter the *value *of the estimated target concentration when using any of the summarization methods, although we can't speculate if it drives the values towards or away from the true value. Also, we note that ArrayInitiative has finally allowed our lab to apply MAS 5.0, dChip and RMA to a BaFL-filtered data set, which has been one our research goals for a while.

## Future Development

In the long term, we intend to develop an open API that will support module development by external programmers for a large number of array types and manufacturers. For example, the research community might create modules that implement a specific strategy for re-defining probe sets (e.g. gene-specific, transcript-specific, exon-specific, tissue-specific, 3'-end specific) or modules that pre-process and remove probes that contain undesirable sequence motifs, such as runs of Gs. Our immediate research goals dictate adding support for Affymetrix SNP and exon arrays, adding support for Agilent human 4 × 44 k arrays, development of a tool to report just the differences between two CDFs, development of a tool to convert between the Affymetrix ASCII and XDA formats and development of a tool to merge two or more different probe set definitions (union, intersection, difference) for the same array type. We also need a variant of the merging tool that can define consensus probe sets among different, but related, platforms. In particular, we have pooled data from adenocarcinoma studies assayed on four versions of the Affymetrix human genome arrays: HG-U95, HG-U133, HG-U133A and HG-U133 Plus 2.0. These arrays share many same-sequence probe pairs, but the names of their parent probe sets and their location on the arrays are different. A consensus merging tool will identify the common probe pairs by their sequence and then group them into biologically relevant probe sets. The probe set identifiers and probe sequences will then be consistent across arrays, differing only in probe coordinates. This would require a custom CDF for each array version, but all of them would consistently measure the same subsequences in each transcript. Finally, we intend to add support for a *difference specification *type, which will allow users to specify a custom CDF as an exact copy of the baseline CDF, except for any explicitly stated differences, most likely useful for those studying only a few genes in great detail.

## Conclusions

ArrayInitiative is for those biological researchers who want to create custom microarray specifications, such as a CDF, without the additional burden of learning the manufacturer's specification file format or learning an API. It provides graphical tools for importing a manufactuer's microarray specification, defining custom versions of a manufacturer's specification, writing array specifications in their standard format or in an easily understandable, non-standard representation. Creating a custom array specification requires only minimal knowledge of a manufacturer's specification standards (file formats and logical rules) and the ability to create a simple delimited or XML file.

The case study illustrated two concepts: the simplicity of using ArrayInitiative to create custom array specifications and how those modified specifications can significantly change summarized expression values. By not being constrained to a specific strategy for re-defining an array specification, ArrayInitiative enables researchers to create new specifications based upon their own requirements. These array specifications might be the result of a new probe-filtering technique or may help to answer a specific biological question. Since it is unclear which re-definition strategies are the best, ArrayInitiative will make it easy to test competing approaches and compare them to the manufacturer's array specification, using established, tested software.

## Availability and requirements

**Project name**: ArrayInitiative

**Project home page**: http://wellerlab.uncc.edu/ArrayInitiative/index.html

**Operating system(s)**: Windows, Linux and Mac OS X

**Programming language**: Python 2.5 - 2.8

**Other requirements**: SQLite, PyQt4

**License**: LGPL

**Restrictions for non-academic use**: None

## List of abbreviations

**API**: application programming interface; **ASCII**: text-based version of Affymetrix CDFs; **BaFL**: biogically applied filters; **CDF**: chip definition file; **MM**: mismatch; **PM**: perfect match; **RAND**: set of randomly selected arrays; **SDK**: software development kit; **SNP**: single nucleotide polymorphism; **XDA**: binary version of Affymetrix CDF.

## Authors' contributions

CO designed and developed ArrayInitiative, designed and carried out the research for the case study and wrote the manuscript. DC and ET developed first-generation code for parsing ASCII and XDA CDFs. KT helped to design the case study. JW supervised the research - helping with the feature requirements for ArrayInitiative and helping to design the case studies - and revised the manuscript. All authors read and approved the final manuscript.

## Supplementary Material

Additional file 1 **ArrayInitiative 1.0**. The first release of ArrayInitiative. Click here for file
