# Supplementary figures and images for: ArrayInitiative - a tool that simplifies creating custom Affymetrix CDFs
Source: BMC Bioinformatics. 2011 May 6;12:136. doi: 10.1186/1471-2105-12-136 (PMC3113937; doi:10.1186/1471-2105-12-136)

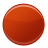

Supplement: Additional file 1 — ArrayInitiative 1.0. The first release of ArrayInitiative. [file 1471-2105-12-136-S1.ZIP › array_initiative_version_1.0/ui/icons/circle_red.png]

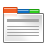

Supplement: Additional file 1 — ArrayInitiative 1.0. The first release of ArrayInitiative. [file 1471-2105-12-136-S1.ZIP › array_initiative_version_1.0/ui/icons/tabs.png]

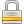

Supplement: Additional file 1 — ArrayInitiative 1.0. The first release of ArrayInitiative. [file 1471-2105-12-136-S1.ZIP › array_initiative_version_1.0/ui/icons/lock.png]

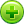

Supplement: Additional file 1 — ArrayInitiative 1.0. The first release of ArrayInitiative. [file 1471-2105-12-136-S1.ZIP › array_initiative_version_1.0/ui/icons/add.png]

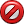

Supplement: Additional file 1 — ArrayInitiative 1.0. The first release of ArrayInitiative. [file 1471-2105-12-136-S1.ZIP › array_initiative_version_1.0/ui/icons/cancel.png]

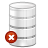

Supplement: Additional file 1 — ArrayInitiative 1.0. The first release of ArrayInitiative. [file 1471-2105-12-136-S1.ZIP › array_initiative_version_1.0/ui/icons/database_remove.png]

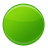

Supplement: Additional file 1 — ArrayInitiative 1.0. The first release of ArrayInitiative. [file 1471-2105-12-136-S1.ZIP › array_initiative_version_1.0/ui/icons/circle_green.png]

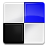

Supplement: Additional file 1 — ArrayInitiative 1.0. The first release of ArrayInitiative. [file 1471-2105-12-136-S1.ZIP › array_initiative_version_1.0/ui/icons/delicious.png]

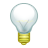

Supplement: Additional file 1 — ArrayInitiative 1.0. The first release of ArrayInitiative. [file 1471-2105-12-136-S1.ZIP › array_initiative_version_1.0/ui/icons/lightbulb.png]

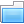

Supplement: Additional file 1 — ArrayInitiative 1.0. The first release of ArrayInitiative. [file 1471-2105-12-136-S1.ZIP › array_initiative_version_1.0/ui/icons/folder.png]

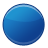

Supplement: Additional file 1 — ArrayInitiative 1.0. The first release of ArrayInitiative. [file 1471-2105-12-136-S1.ZIP › array_initiative_version_1.0/ui/icons/circle_blue.png]

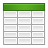

Supplement: Additional file 1 — ArrayInitiative 1.0. The first release of ArrayInitiative. [file 1471-2105-12-136-S1.ZIP › array_initiative_version_1.0/ui/icons/table_green.png]

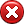

Supplement: Additional file 1 — ArrayInitiative 1.0. The first release of ArrayInitiative. [file 1471-2105-12-136-S1.ZIP › array_initiative_version_1.0/ui/icons/close.png]

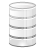

Supplement: Additional file 1 — ArrayInitiative 1.0. The first release of ArrayInitiative. [file 1471-2105-12-136-S1.ZIP › array_initiative_version_1.0/ui/icons/database.png]

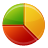

Supplement: Additional file 1 — ArrayInitiative 1.0. The first release of ArrayInitiative. [file 1471-2105-12-136-S1.ZIP › array_initiative_version_1.0/ui/icons/pie_chart.png]

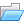

Supplement: Additional file 1 — ArrayInitiative 1.0. The first release of ArrayInitiative. [file 1471-2105-12-136-S1.ZIP › array_initiative_version_1.0/ui/icons/folder_open.png]

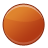

Supplement: Additional file 1 — ArrayInitiative 1.0. The first release of ArrayInitiative. [file 1471-2105-12-136-S1.ZIP › array_initiative_version_1.0/ui/icons/circle_orange.png]

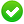

Supplement: Additional file 1 — ArrayInitiative 1.0. The first release of ArrayInitiative. [file 1471-2105-12-136-S1.ZIP › array_initiative_version_1.0/ui/icons/check_mark.png]

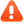

Supplement: Additional file 1 — ArrayInitiative 1.0. The first release of ArrayInitiative. [file 1471-2105-12-136-S1.ZIP › array_initiative_version_1.0/ui/icons/alert.png]

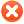

Supplement: Additional file 1 — ArrayInitiative 1.0. The first release of ArrayInitiative. [file 1471-2105-12-136-S1.ZIP › array_initiative_version_1.0/ui/icons/x.png]
